# 429. SARS-CoV-2 Fecal Shedding Dynamics Among People with Symptomatic COVID-19

**DOI:** 10.1093/ofid/ofae631.143

**Published:** 2025-01-29

**Authors:** Tiffany Hink, Katelyn L Parrish, Kimberly Reske, Olivia G Arter, Katheryn Ney, Victoria Joachimstaler, Brittany L Roemmich, Sydney M Lawless, Meghan L Brown, David Wang, Yuxiong Liu, Srikanth Singamaneni, Christopher W Farnsworth, Bijal Parikh, Erik Dubberke

**Affiliations:** Washington University, St. Louis, Missouri; Washington University School of Medicine, Saint Louis, Missouri; Washington University, St. Louis, Missouri; Washington University in St. Louis, St. Louis, Missouri; Washington University School of Medicine, Saint Louis, Missouri; Washington University School of Medicine, Saint Louis, Missouri; Washington University School of Medicine, Saint Louis, Missouri; Washington University School of Medicine, Saint Louis, Missouri; Washington University School of Medicine, Saint Louis, Missouri; Washington University School of Medicine, Saint Louis, Missouri; Washington University, St. Louis, Missouri; Washington University, St. Louis, Missouri; Washington University School of Medicine, Saint Louis, Missouri; Washington University School of Medicine, Saint Louis, Missouri; Washington University School of Medicine, Saint Louis, Missouri

## Abstract

**Background:**

Wastewater surveillance (WWS) has become a vital tool to identify SARS-CoV-2 (SCV2) trends in the community and emerging variants. A limitation to interpretation of WWS SCV2 data is an incomplete understanding of SCV2 RNA shedding dynamics in feces. The SCV2 Fecal Shedding Dynamics (FLuSHED) study sought to quantify SCV2 RNA fecal shedding in order to improve WWS data interpretation.

**Table 1.**
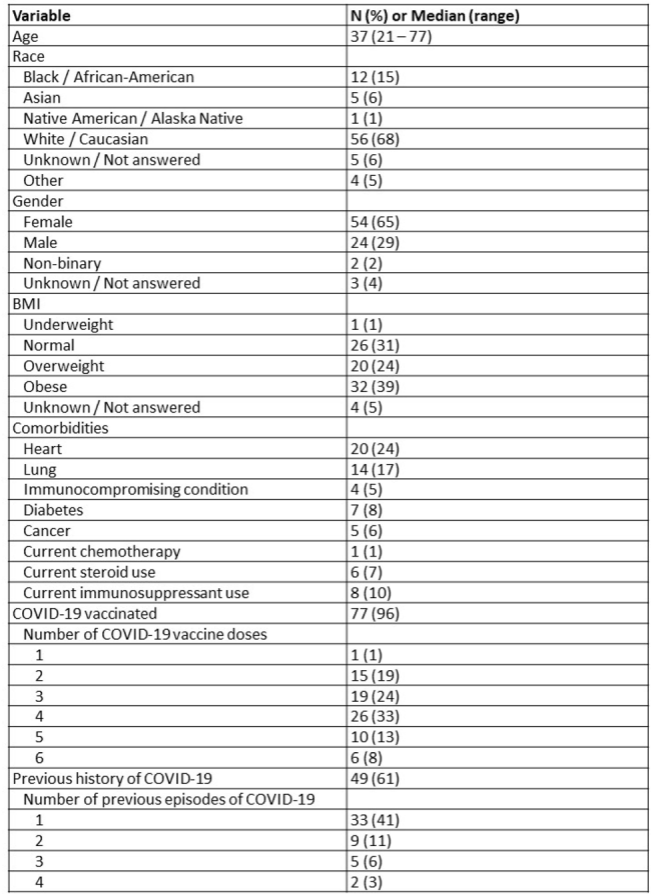
Demographics of Study Population (N=83 subjects who submitted ≥1 specimen, including enrollment stool)

**Methods:**

FLuSHED is a prospective cohort study conducted in St. Louis, MO. Individuals who tested positive for SCV2 within 7 days of symptoms onset were enrolled. Participants submitted stool specimens and nasal swabs at enrollment and weekly until day 35-post enrollment and completed weekly surveys regarding COVID-19 symptoms, treatments, healthcare visits, and demographics/medical history. Dried blood spots and whole blood were collected at enrollment and day 35 to evaluate SCV2 antibody response; NP swabs were tested for SCV2 antigen by plasmonic fluor-linked immunosorbent assay (p-FLISA) and RNA by PCR. Stool was tested for RNA by PCR.

**Table 2.**
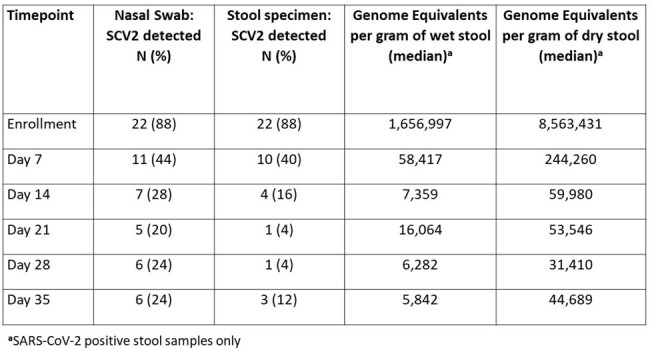
Detection of SCV2 RNA via PCR in Nasal Swab and Stool Specimens (n=25 full sets of specimens)

**Results:**

99 participants have been enrolled in FLuSHED to date from March 2023 to April 2024; 83 submitted at least 1 stool specimen (Table 1). Median time from symptom onset to enrollment was 3.5 days; 96% of participants reported being vaccinated against SCV2 (Table 1). PCR to date has been performed on 25 complete patient specimen sets (Table 2). SCV2 was detected in 88% of enrollment stool specimens, 40% of day 7 stool specimens, and only intermittently after day 7 post-enrollment. SCV2 was detected in the day 35 stool of one participant despite the patient’s day 14, day 21, and day 28 stools being SCV2 negative. At enrollment >90% had anti-S antibodies and 62% had anti-N antibodies; at day 35 the prevalence of anti-N antibodies increased to 93% (Table 4). N protein was detected in 65/84 (77%) NP swabs by p-FLISA, with a limit-of-detection of 8pg/mL (Figure 1).

**Table 3.**
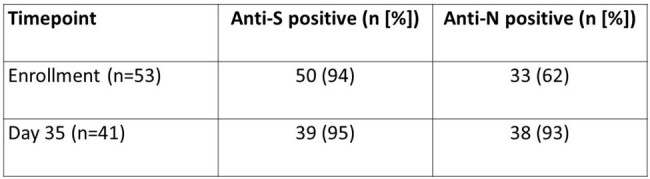
Anti-S and Anti-N antibody positive at enrollment and day 35

**Conclusion:**

This study remains ongoing, but analyses from the first year of enrollment and follow-up indicate that SCV2 shedding in stool was common during and shortly after acute SCV2 illness. Later shedding of SCV2 virus is less common, but may continue to contribute to presence of SCV2 RNA in wastewater.

**Figure 1.**
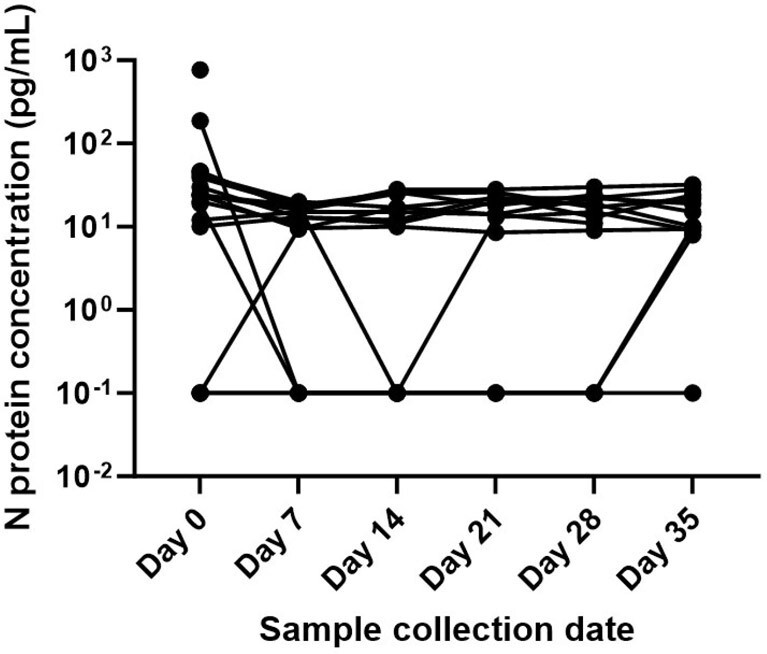
N protein p-FLISA test detection of SARS-CoV-2 virus

**Disclosures:**

**Srikanth Singamaneni, PhD**, Auragent Bioscience, LLC: Inventor on a pending patent related to plasmonic-fluor technology (licensed by Washington Univ in St. Louis to Auragent Bioscience, LLC|Auragent Bioscience, LLC: Ownership Interest **Christopher W. Farnsworth, PhD**, Abbott: Advisor/Consultant|Abbott: Grant/Research Support|Abbott: Honoraria|BD: Advisor/Consultant|Beckman Coulter: Grant/Research Support|Bluejay Diagnostics: Grant/Research Support|Roche: Grant/Research Support|Roche: Honoraria|Sebia: Grant/Research Support|Siemens: Grant/Research Support|Werfen: Advisor/Consultant|Werfen: Honoraria **Bijal Parikh, MD, PhD**, Cepheid: Honoraria **Erik Dubberke, MD, MSPH**, AstraZeneca: Advisor/Consultant|AstraZeneca: Grant/Research Support|Ferring: Advisor/Consultant|Ferring: Grant/Research Support|Merck: Advisor/Consultant|Pfizer: Grant/Research Support|Recursion: Advisor/Consultant|Seres Therapeutics: Advisor/Consultant|Theriva Biologics: Grant/Research Support|Vedanta: Grant/Research Support

